# Fistulous empyema due to bronchopulmonary laceration with a misintubated nasogastric tube: a case report

**DOI:** 10.1186/s44215-025-00201-w

**Published:** 2025-03-17

**Authors:** Ryosuke Matsuda, Yuuki Kou, Yuya Kogita, Yasushi Sakamaki

**Affiliations:** Department of Thoracic Surgery, Osaka Keisatsu Hospital, 2-6-40 Karasugatsuji, Tennoji-Ku, Osaka, 543-8922 Japan

**Keywords:** Fistulous empyema, NGT misplacement

## Abstract

**Background:**

Nasogastric tube (NGT) misinsertion into the airway can sometimes cause penetrating trauma, resulting in pneumothorax or empyema which can lead to critical respiratory failure if not promptly recognized. Elderly patients with a diminished cough reflex and impaired communication are particularly vulnerable to NGT misinsertion. We report a case of fistulous empyema caused by tube feeding through an NGT that was misinserted into the airway and penetrated into the pleural cavity.

**Case presentation:**

An 82-year-old bedridden woman with severe disability and a medical history of intracerebral hemorrhage was transferred to our department because of acute respiratory failure a day after her NGT was replaced at the referring hospital. During the 19 h between NGT replacement and the first observation of respiratory failure, tube feedings were administered twice via the new NGT. Computed tomography revealed NGT misinsertion into the left lower lobe bronchus and massive liquid accumulation with pneumothorax in the left pleural cavity, suggesting a penetrating bronchopulmonary trauma. After the patient was transferred to our hospital, a chest tube was inserted immediately to drain the contents of the tube feeding that had accumulated in the pleural space. Several days later, surgery was performed to irrigate the empyema cavity and repair the laceration. The postoperative course was uneventful, and the patient returned to the referring hospital.

**Conclusions:**

Our case highlights the importance of careful NGT insertion and recognizing misinsertion by radiological findings to avoid severe airway complications, particularly in elderly and neurologically impaired patients.

## Background

Nasogastric tube (NGT) insertion is a common clinical procedure, but it requires careful attention, as NGT misplacement is often overlooked, particularly in elderly patients with disabilities who have a weakened cough reflex and impaired communication. Such incidents can lead to fatal outcomes if NGT misinsertion is not recognized. We herein report a case of fistulous empyema caused by feeding through an NGT that was misinserted into the airway and penetrated the bronchus and the lung into the pleural cavity.

## Case presentation

**Fig. 1:**
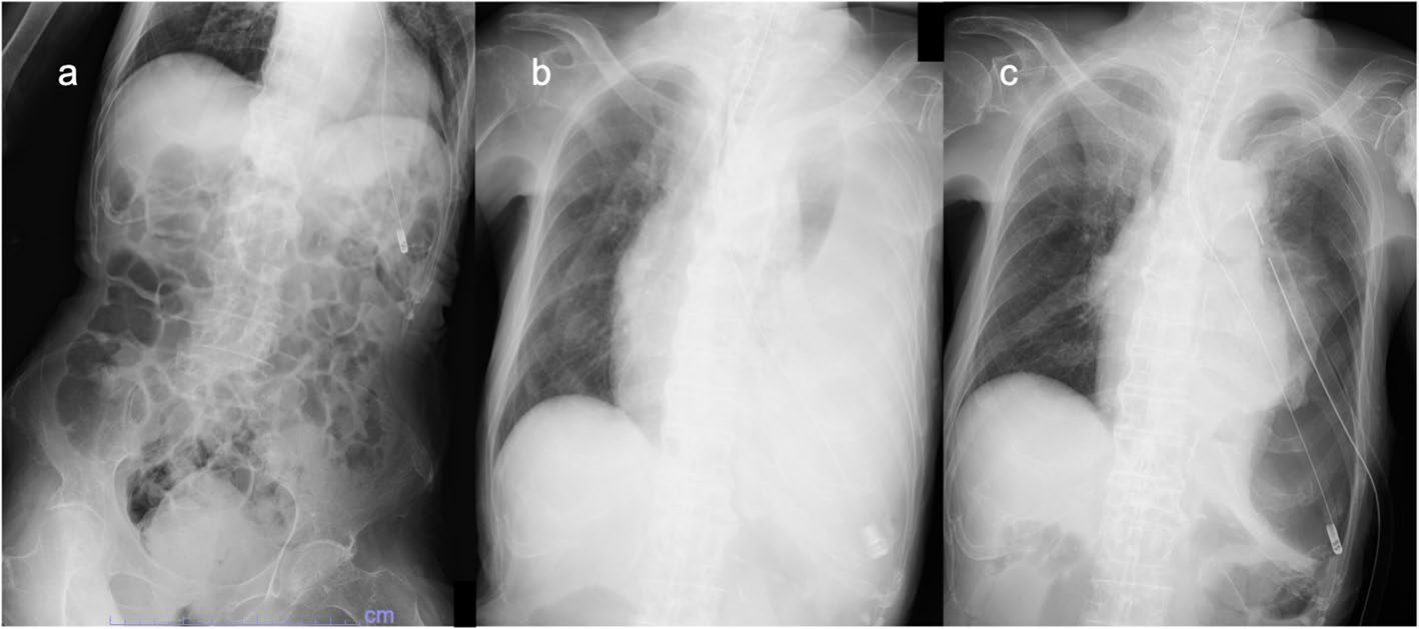
Chest X-ray images. **a** Immediately after the nasogastric tube (NGT) replacement. **b** Decreased radiolucency in the entire left lung field 19 h after the NGT replacement. **c** Misinserted NGT also shown after pleural space drainage at our department

**Fig. 2:**
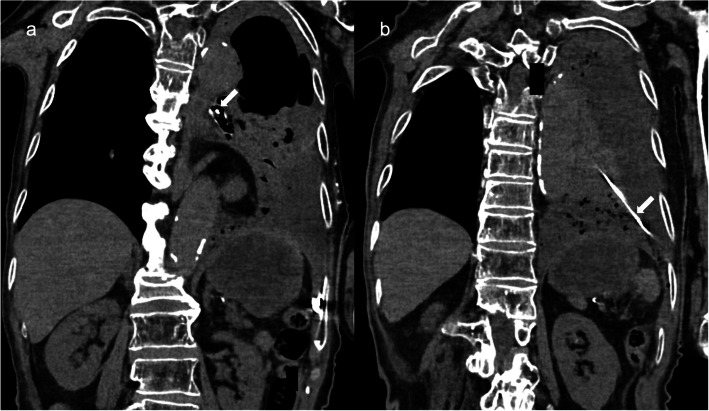
Chest CT images. The nasogastric tube passed through (**a**) the left main bronchus and (**b**) into the pleural cavity (arrows)

**Fig. 3:**
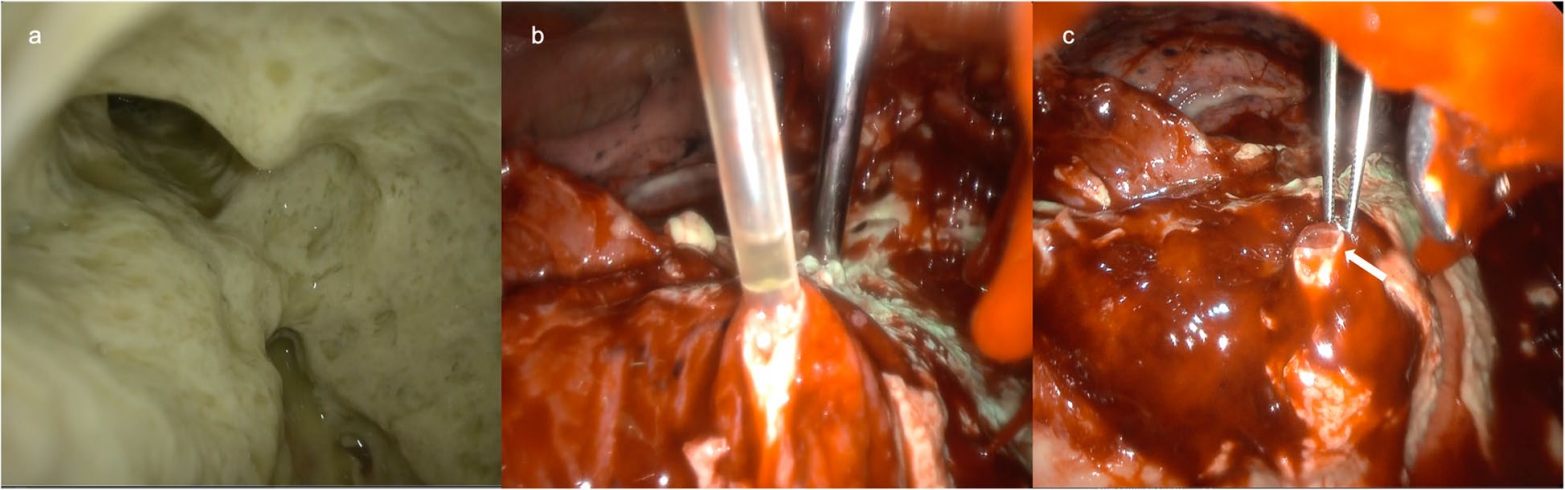
Intraoperative findings. **a** Purulent material in the empyema cavity. **b** The NGT protruding into the pleural cavity after curettage and irrigation. **c** Fistula orifice (arrow)

An 82-year-old woman who was bedridden, unable to express her intent, and completely dependent on others due to sequelae of intracerebral hemorrhage was transferred to our hospital because of a left-sided fistulous empyema requiring surgical treatment. Upon transfer, the patient was reportedly found to have developed acute respiratory failure 19 h after her last NGT replacement at the referring hospital. During the interval between NGT replacement and the initial recognition of respiratory distress (oxygen saturation: 80%), 400 mL of enteral nutrition was administered twice through the newly placed NGT. The routine confirmatory X-ray taken immediately after NGT replacement primarily showed the abdominal region, with minimal chest coverage (Fig. 1a), leading to unrecognized misplacement at that time. However, shortly after the patient’s condition deteriorated, chest X-ray (CXR) showed a remarkable air space and a significant reduction in radiolucency in the left lung field, suggesting fluid collection in the left pleural cavity and associated lung collapse (Fig. 1b). Computed tomography (CT) revealed that a misinserted NGT into the trachea had passed through the left lower lobe bronchus and advanced into the left pleural cavity with pneumothorax and massive fluid accumulation (Fig. 2). As the patient’s son had already opted for a do-not-attempt-resuscitation policy and initially declined surgery, our initial treatment was limited to thoracic drainage and antibiotics (sulbactam/ampicillin 3 g every 8 h) for the pleural infection caused by multidrug-susceptible staphylococcus aureus. However, her son finally agreed to definitive surgery based on repeated informed consent, confirming that the patient’s condition was stable with conservative treatment. On the sixth day after transfer to our hospital, definitive surgery was performed to control the infection and air leakage. Through a 6-cm skin incision along the sixth intercostal space, the empyema cavity was entered, and the NGT penetrating the left lower lobe was encountered. After curettage and irrigation of the empyema cavity, the NGT was shortened by cutting the tip and removed by pulling it out through the nose, which revealed the orifice of the fistula measuring approximately 5 mm in diameter (Fig. 3). The bronchopleural fistula was repaired with direct suture closure using 4–0 polypropylene, reinforced by a free flap of the serratus anterior muscle. The postoperative course was uneventful, and the patient returned to the referring hospital on postoperative day 8. To date, no signs of relapsing empyema have been detected.

## Discussion and conclusions

Generally, NGT insertion is considered a safe and simple procedure in the routine clinical setting. However, NGT misinsertion, although rare, does occur and can sometimes result in serious complications. If an NGT enters the airway, or even if normally inserted into the digestive tract, it can cause serious complications such as pneumothorax, subcutaneous emphysema, diaphragmatic injury, esophageal perforation, and empyema, with the incidence rates ranging from 0.3 to 15% [1]. Rassias et al. [2] conducted a prospective study on complications of NGT insertion and reported an airway misplacement rate of 2% and a mortality rate of 0.3% in 740 cases. Of these complications, empyema is extremely rare, with only 10 reported cases of empyema caused by NGT misplacement in a PubMed search for the English-language literature published from 1981 to 2024 using the keywords “empyema,” “pyothorax,” “nasogastric tube,” and “enteral tube feeding” [1, 3–11]. Haas et al. [12] coined the term “nutrothorax” to describe the iatrogenic pleural effusion caused by enteral nutrients administered through a misplaced NGT, as seen in our case. To our knowledge, eight cases of empyema, including our case, were caused by nutrothorax, and the other two were caused by the administration of activated charcoal to treat a drug overdose. Four of the 11 cases were fatal. Details of the reported cases are shown in Table 1.

**Table 1:**
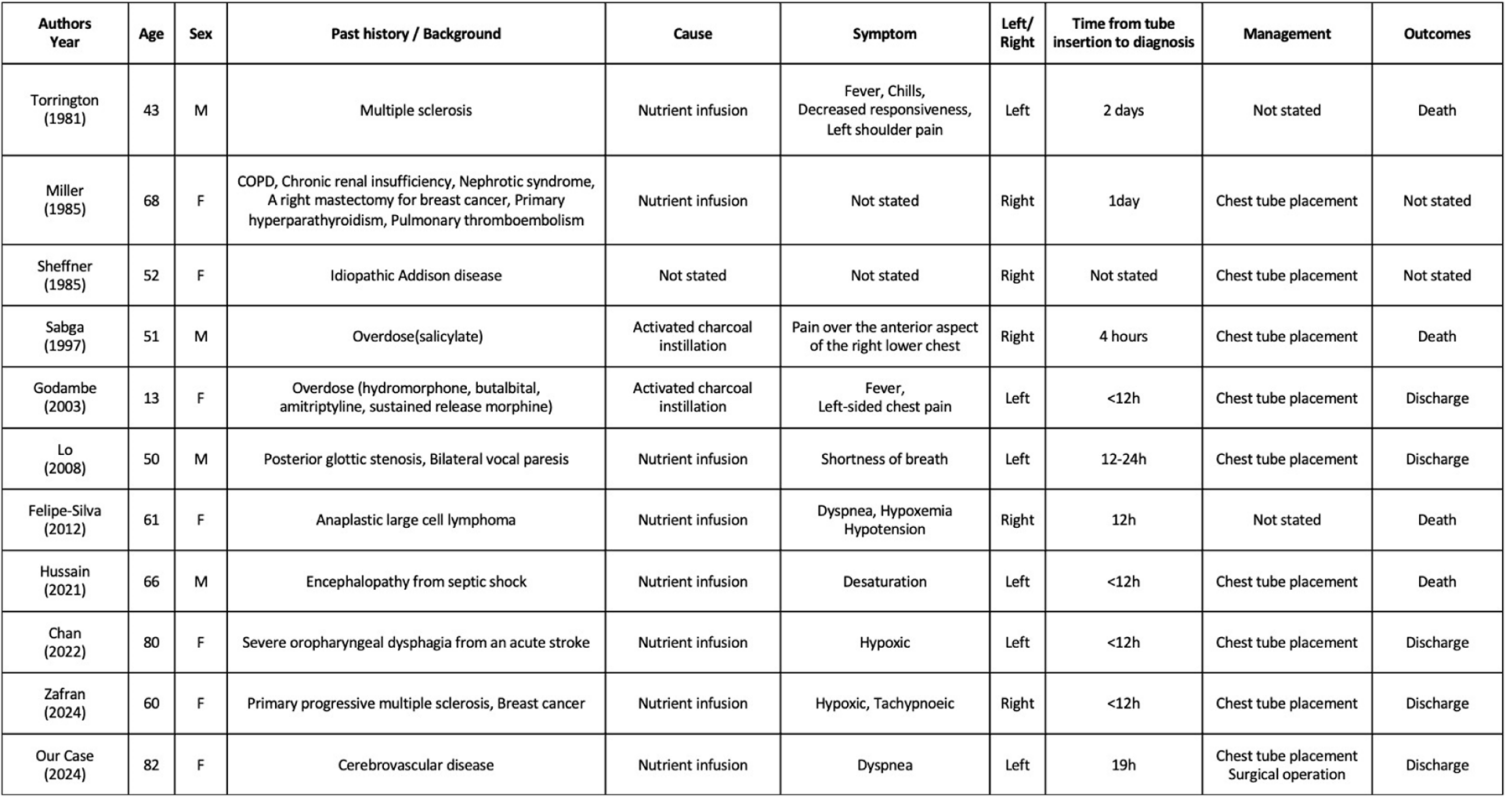
Previously reported cases of fistulous empyema resulting from misplaced nasogastric tube

Cases of misinserted NGTs were predominantly seen in patients with dysphagia, impaired cough or gag reflexes, or communication disorders, suggesting that elderly or pediatric patients with underlying neurological disorders are at a higher risk for NGT misplacement. Dees et al. [13] also highlighted an increased risk in patients with esophageal strictures, comatose, severe craniofacial trauma, and oral intubation. Halloran et al. [14] reported that a smaller internal diameter of the NGT, leading to increased flexibility, was associated with a higher incidence of airway misplacement. Small-bore tubes, which are more flexible than large-bore tubes and designed for the administration of nutrients or medications, were associated with a higher rate of misinsertion into the airway compared with large-bore tubes used for suction from the gastric lumen, which are stiffer and more likely to maintain the correct trajectory but carry a higher risk of mechanical injury to the nasopharynx and esophagus. The possible mechanisms in our case included the stiffness of the small-bore NGT, decreased integrity of the bronchial and pleural tissues due to advanced age, impaired consciousness, and diminished cough reflex. These factors may have contributed to the unintentional penetration into the thoracic cavity.

Several extraordinary verification methods have been recommended to mitigate the incidence of NGT misinsertion [14–16]. Halloran et al. [14] described a method to confirm proper placement by taking a first x-ray after advancing the tube 30 cm up the nose to confirm insertion into the esophagus and a second x-ray after final placement. Lateral chest radiography has also been suggested as a more straightforward and effective method for detecting NGT misplacement [10]. Other recent studies have demonstrated the efficacy and safety of video-assisted laryngoscopy or a camera-equipped tube to directly visualize anatomical landmarks during NGT insertion [15, 16]. Optimizing radiographic coverage is crucial to detect NGT misplacement and prevent associated complications. As demonstrated in this case, inadequate coverage during confirmatory radiography can lead to unrecognized complications. Such limited coverage can lead to poor visualization of the trachea, bronchi, and pleural cavities, resulting in delayed recognition of NGT misplacement. We recommend that a chest radiograph be obtained immediately after proper positioning of the NGT to fully visualize the thoracic cavity and adjacent structures. To prevent repeated failures, consideration should be given to implementing protocols for systematic imaging after NGT placement and providing additional training to medical staff in the interpretation of NGT-related radiographs.

For fistulous empyema, Lu et al. [17] emphasized surgical treatment according to the severity of each patient’s empyema. In their series, severe infection cases characterized by highly viscous purulent discharge were considered critical and often necessitated staged surgery, starting with a fenestration procedure to control infection by promoting sterilization of the cavity, and finally closing the cavity with a pedicled muscle flap [17]. By contrast, cases with less viscous discharge were considered mild and curable by a single-stage surgery. In this report, although the viscosity was roughly evaluated based on intraoperative observation, we defined the severity of infection according to the viscosity of the purulent discharge, as proposed by Lu et al. [17]. Based on this definition, we determined that our patient had a relatively mild infection and that we could perform primary closure in a single surgical procedure. According to general principles, whether a single-stage procedure with primary closure of the bronchopleural fistula (BPF) or a staged procedure including temporary chest wall fenestration is more appropriate must be decided on a case-by-case basis, depending on the availability of direct sutures or native tissue packing for BPF closure. In severely infected pleural cavities, primary closure is almost impossible because of a very high probability of suture site dehiscence due to the fragility of the tissues affected by inflammation and the prevention of healthy granulation between the fistula and the translocated tissue (muscular or omental flap). In our case, the empyema was localized with a relatively mild infection and the peripheral bronchial perforation could be covered by suturing en masses with the surrounding lung tissue, so irrigation of the empyema cavity with subsequent repair of the fistula was successfully performed in a single surgery, resulting in a favorable outcome.

In conclusion, NGT misplacement into the airway is a critical medical error that can lead to fatal outcomes if not promptly recognized. As described here, careful checks during and immediately after insertion are mandatory, especially for high-risk patients. If complications are suspected, early diagnosis of the complication followed by prompt treatment, including surgery, is essential to improve patient outcomes.

## Data Availability

Not applicable.

## Abbreviations

NGT: Nasogastric tube
CXR: Chest X-ray
CT: Computed tomography
BPF: Bronchopleural fistula
